# Supplementary material for: A Concerted Action of UBA5 C-Terminal Unstructured Regions Is Important for Transfer of Activated UFM1 to UFC1
Source: Int J Mol Sci. 2021 Jul 9;22(14):7390. doi: 10.3390/ijms22147390 (PMC8304084; doi:10.3390/ijms22147390)
Supplement: Supplementary file 1 [file ijms-22-07390-s001.zip › ijms-1284146-supplementary.pdf]

Suppementary Material

A Concerted Action of UBA5 C-Terminal Unstructured Regions Is Important for Transfer of Activated UFM1 to UFC1

Nicole Wesch [1], Frank Löhr [1], Natalia Rogova [1], Volker Dötsch [1,2,*] and Vladimir V. Rogov [1,2,3,*]

[1] Institute of Biophysical Chemistry and Center for Biomolecular Magnetic Resonance, Goethe-University Frankfurt, 60438 Frankfurt am Main, Germany; Wesch@bpc.uni-frankfurt.de (N.W.); Murph@bpc.uni-frankfurt.de (F.L.); Rogova@bpc.uni-frankfurt.de (N.R.)

[2] Structural Genomics Consortium, Buchmann Institute for Life Sciences, Goethe-University Frankfurt, 60438 Frankfurt am Main, Germany

[3] Institute of Pharmaceutical Chemistry, Goethe-University Frankfurt, 60438 Frankfurt am Main, Germany

* Correspondence: vdoetsch@em.uni-frankfurt.de (V.D.); rogov@pharmchem.uni-frankfurt.de (V.V.R.)

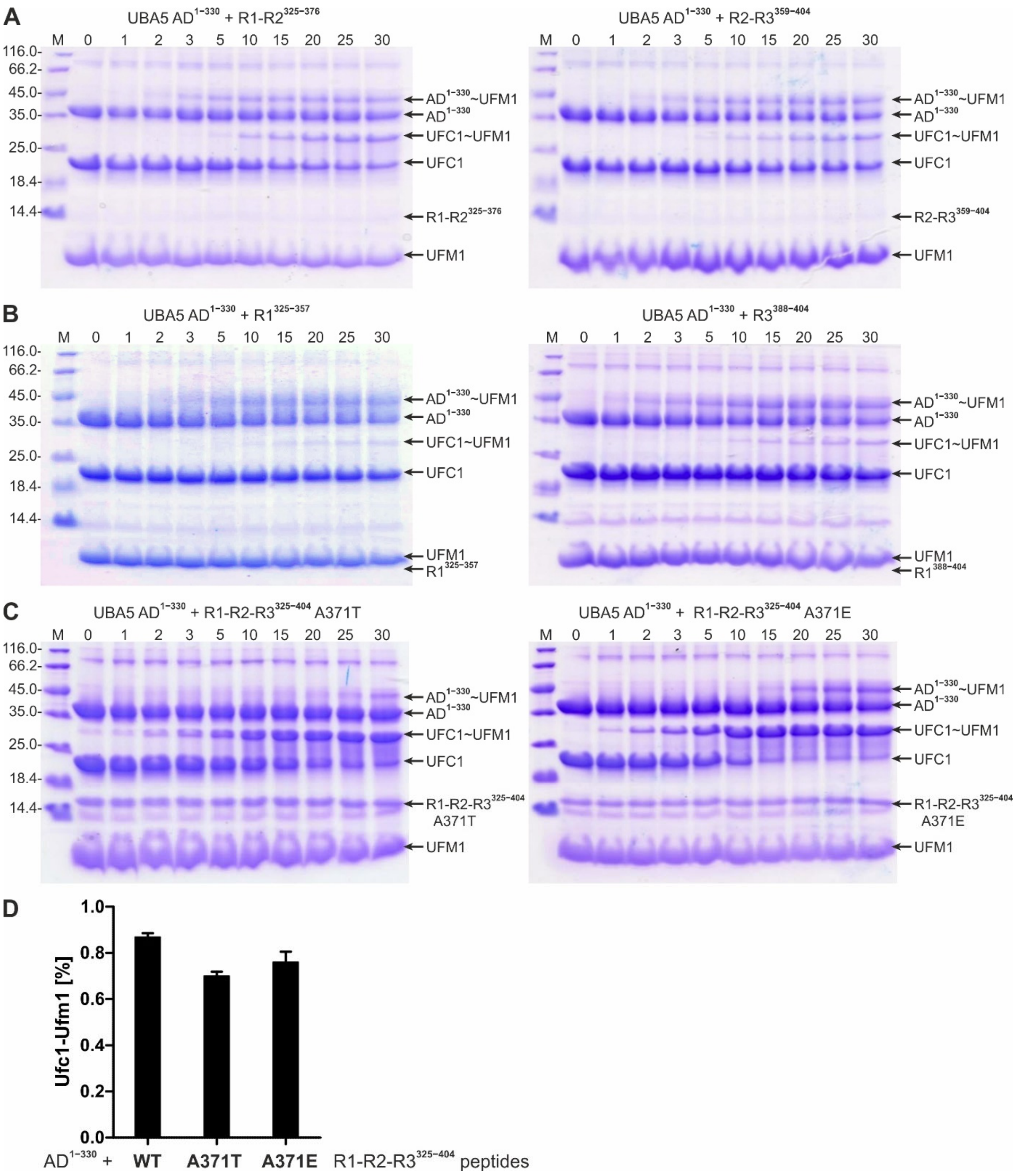

Figure S1. UFC1 ufmylation mediated by different UBA5 constructs. Gel electrophoresis of ufmylation assays including UBA5 adenylation domain (AD^{1-330}) and C-terminal peptides R1-$R2^{32-376}$ or R2-$R3^{359-404}$ (**A**), $R1^{325-357}$ or $R3^{381-404W}$ (**B**) and R1-R2-$R3^{325-404}$ A371T or R1-R2-$R3^{325-404}$ A371E (**C**) Ufmylation was tracked over 30 minutes. Corresponding protein bands are labelled on the right side. (**D**) Quantification of UFC1~UFM1 conjugate formation in reactions with UBA5 AD^{1-330} and wild type, A371T or A371E mutated R1-R2-$R3^{325-404}$ peptides after 10 minutes. For quantification of conjugated and not conjugated UFC1 coloc2 software implemented in ImageJ was used.

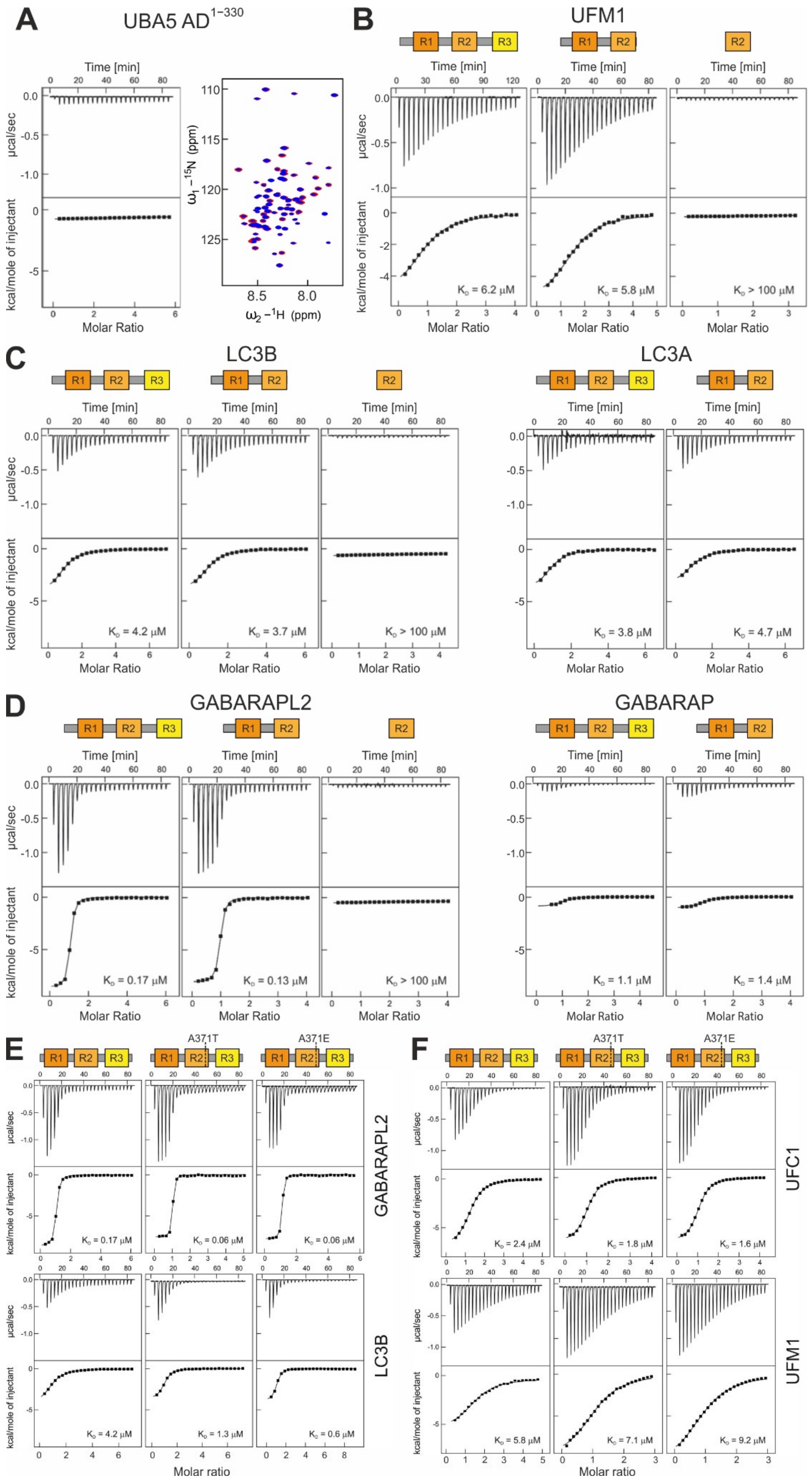

Figure S2. Interaction between UBA5 C-terminal constructs and UBA5 interacting proteins observed by ITC experiments. (**A**) No interactions between UBA5 adenylation domain and C-terminal parts were observed by ITC (left plot) and NMR (right plot) titration experiments. In ITC experiment, R1-R2-R3$^{325–404}$ peptide was titrated into an AD$^{1–330}$ solution. In NMR

experiment, non-labelled AD^{1-330} construct was added to the ^{15}N-labelled R1-R2-$R3^{325-404}$ peptide to a 2-fold molar excess. Representative area of [^{15}N,^{1}H] TROSY-HSQC spectra recorded at 900 MHz for R1-R2-$R3^{325-404}$ in free form (red contours) and in presence of AD^{1-330} (blue contours) are overlaid. (**B-D**) ITC titrations of the different C-terminal UBA5 peptides (graphically visualized above the corresponding titration profiles) and UFM1 (**B**), LC3A or LC3B (**C**) and GABARAP or GABARAPL2 (**D**). (**E-F**) ITC titration profiles for interaction between R1-R2-$R3^{325-404}$ peptides containing A371T or A371E mutations and GABARAPL2 or LC3B (**E**), UFC1 or UFM1 (**F**). The upper graphs display the raw heat data; the lower graphs show the integrated heat per titration steps (black squares) with best-fit curve (line). The used peptides are graphically visualized above the corresponding titration profiles. K_D values are indicated.

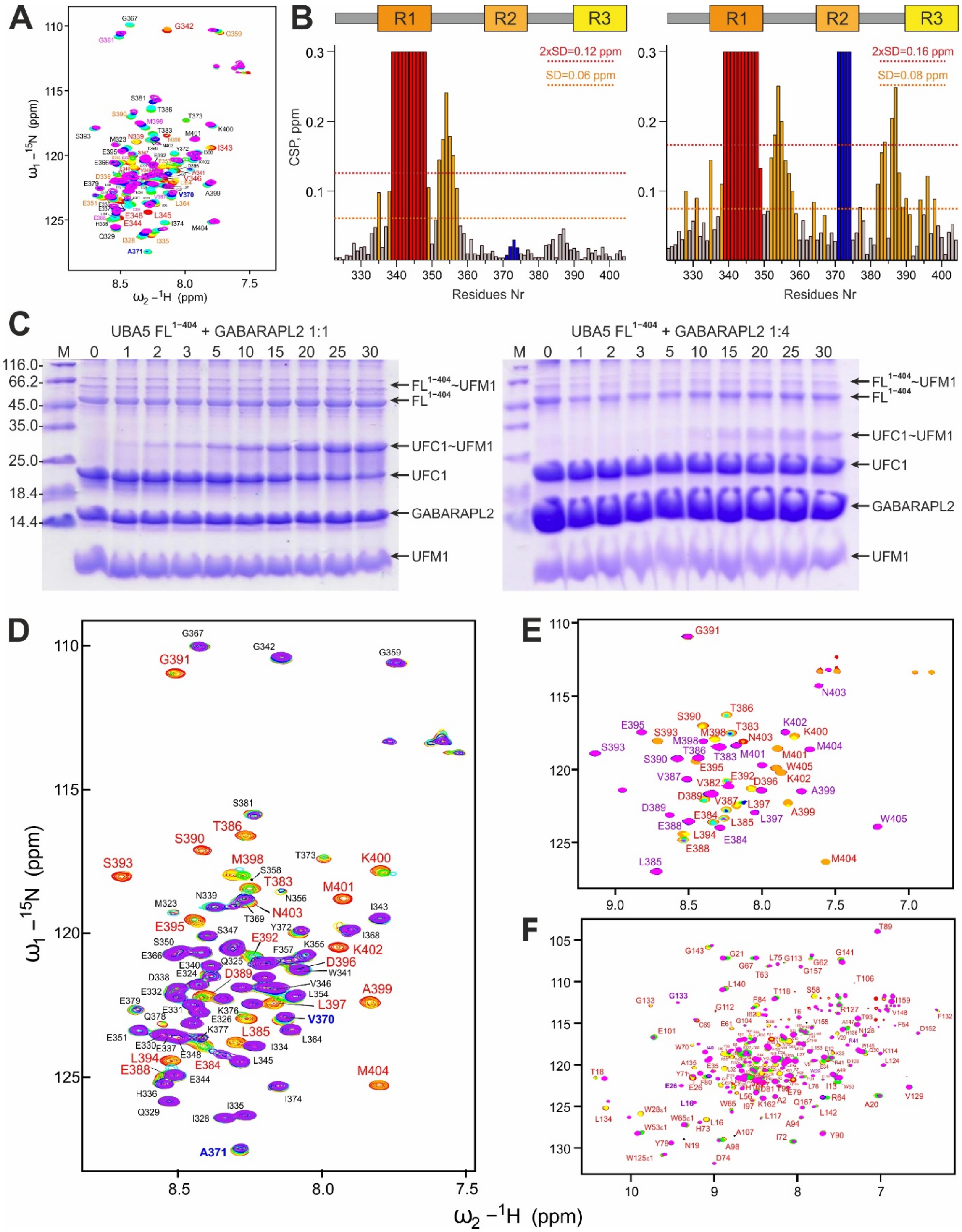

Figure S3. Interactions between UBA5 C-terminal peptides and UBA5-interacting proteins observed by NMR titration experiments. (**A**) NMR titration of ^{15}N-labelled R1-R2-R3$^{325–404}$ peptide with unlabelled GABARAPL2. An overlay of the representative areas of the [^{15}N,^{1}H] TROSY-HSQC spectra recorded at 500 MHz is presented. The titration steps are indicated with a rainbow colour code from free R1-R2-R3$^{325–404}$ peptide (red) to saturation with GABARAPL2 (purple; molar ratio 1:4). The respective CSP analysis is presented in (**B**) (at ratio 1:1, left; and ratio 1:4, right) as a bar diagram. Bars for disappearing peaks are labelled red while significant CSPs (>1xSD) are coloured orange; peaks which CSPs <1xSD are coloured grey. Residues within UBA5 R2 region near the A371 are labelled blue. (**C**) Ufmylation competition assays between UFM1 and GABARAPL2. GABARAPL2 was added in a stoichiometry of 1:1 (left) and 1:4 (right) to the ufmylation reaction mix. Ufmylation was tracked over 30 minutes. Corresponding protein bands are labelled on the right side. (**D**) Overlay of full-size [^{15}N,^{1}H] TROSY-HSQC spectra recorded at 500 MHz upon titration of ^{15}N-labeled R1-R2-R3$^{325–404}$ peptide with non-labelled UFC1. (**E**) Overlay of full-size [^{15}N,^{1}H] TROSY-HSQC spectra recorded at 800 MHz upon titration

of ^{15}N-labeled R3$^{381–404W}$ peptide with non-labelled UFC1. (**F**) Overlay of full-size [^{15}N,^{1}H] TROSY-HSQC spectra recorded at 950 MHz upon titration of ^{15}N-labeled UFC1 protein with non-labelled R3$^{381–404W}$ peptide. The molar ratios and colour codes are the same as in Figure 2B-E.

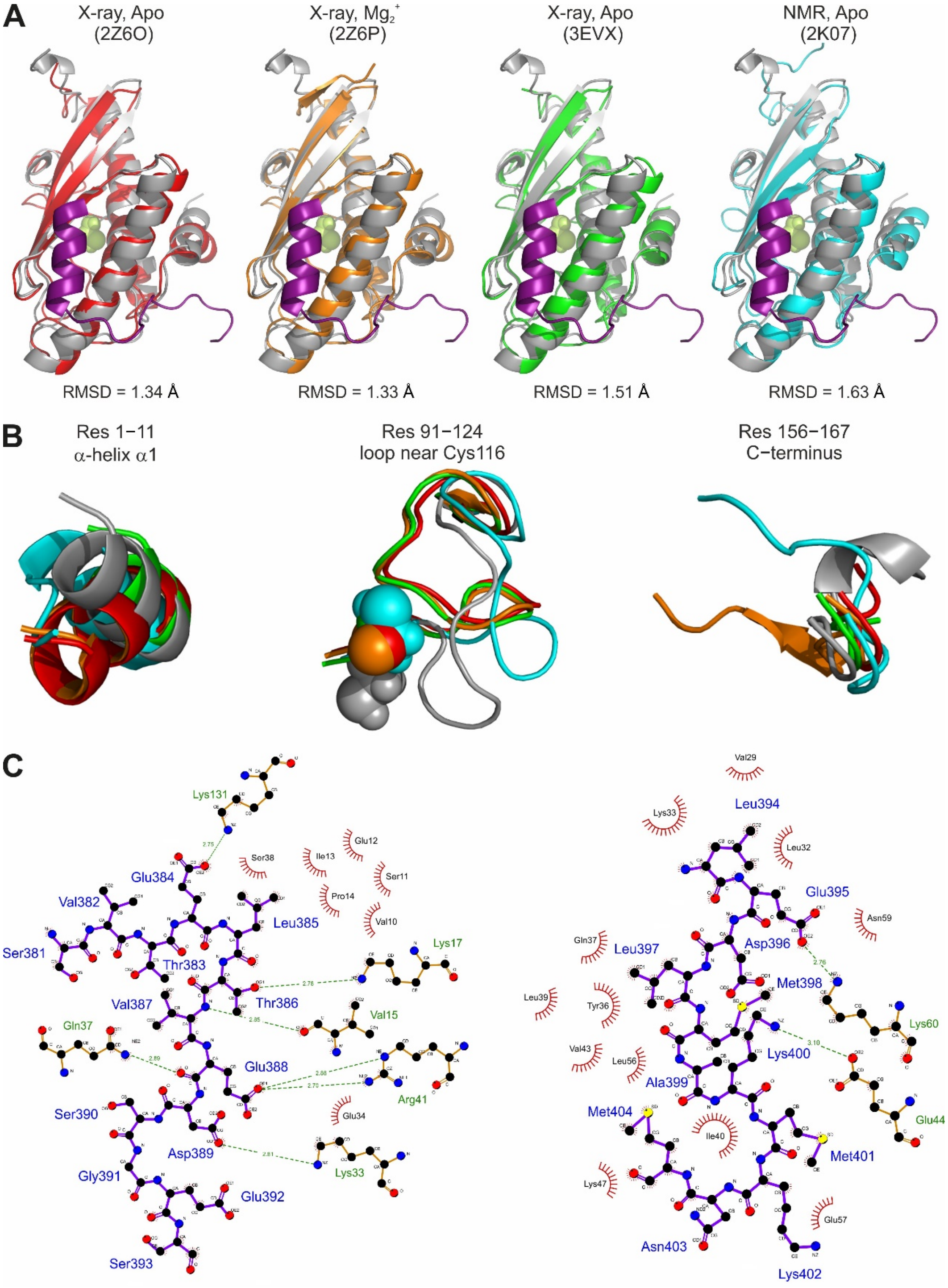

Figure S4. Structural features of UFC1 in complex with UBA5 R3 peptide. (**A**) Superimposition of the UFC1:R3$^{381–404W}$ complex structure calculated in this work (grey) and previously published X-ray (2Z6O (red), 2Z6P (orange), 3EVX (green)) and NMR free (2K07 (cyan)) UFC1 structures [1,2]. The R3$^{381–404W}$ peptide is shown in purple. (**B**) Detailed view on most significant differences observed in the orientation of the N-terminal α-helix α1 (residues 1–11, left plot), the flexible loop near the active-cite cysteine 116 (residues 91–124, middle plot) and conformation of the C-terminal UFC1 part (residues 156–167, right plot). (**C**) Intermolecular contacts evaluated by LigPlot software for representative UFC1:R3$^{381–404W}$ conformer.

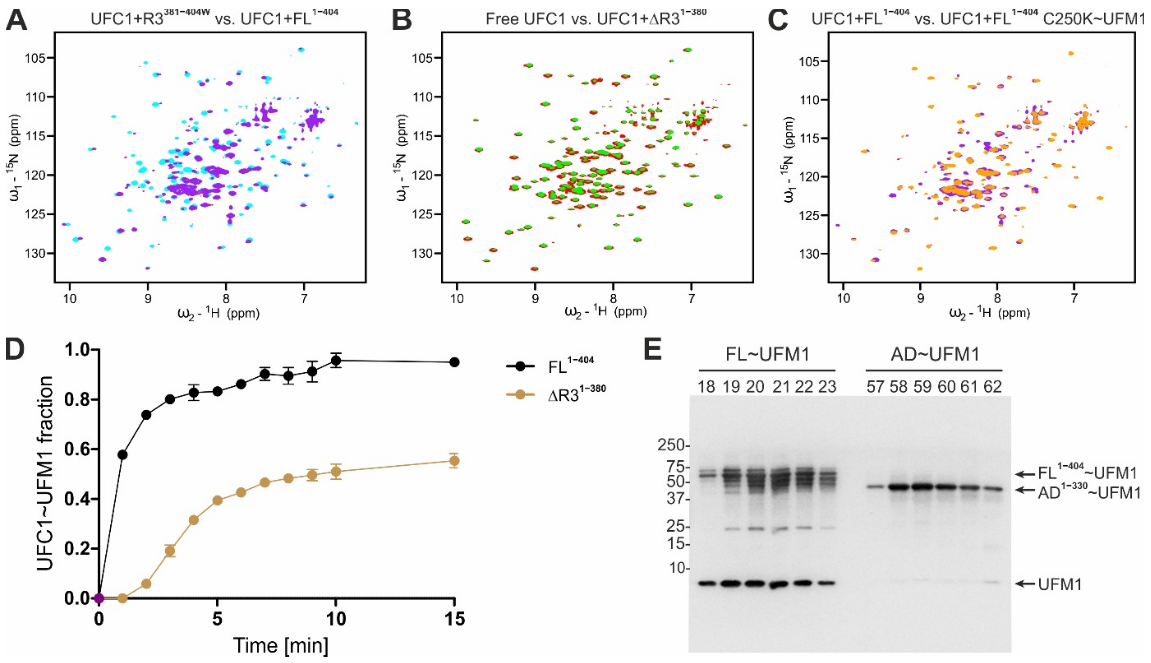

Figure S5. UFC1 binding to the full length UBA5 and UBA5~UFM1 conjugate. (**A**) Overlay of the [^{15}N,^{1}H] TROSY-HSQC spectra recorded at 950 MHz for ^{15}N-labelled UFC1 bound to non-labelled R3$^{381-404W}$ (cyan) and FL$^{1-404}$ (purple). Note that the overall molecular mass of the UFC1:FL$^{1-404}$ complex is relatively high, therefore, NMR spectroscopy did not allow us to investigate this site in details. (**B**) Overlay of the [^{15}N,^{1}H] TROSY-HSQC spectra (950 MHz) for free ^{15}N-labelled UFC1 (red) and UFC1 in presence of 2-times molar excess of R3-depleted UBA5 (ΔR3$^{1-380}$, green). (**C**) Overlay of the [^{15}N,^{1}H] TROSY-HSQC spectra (950 MHz) for ^{15}N-labelled UFC1 in presence of 2-times molar excess of FL$^{1-404}$ (purple) and FL$^{1-404}$ C250K~UFM1 (orange). (**D**) Ufmylation assays tracked over 15 minutes using UBA5 FL$^{1-404}$ or ΔR3$^{1-380}$ constructs. All assays were done as triplicates. Evaluation of UFC1~UFM1 conjugate was done via western blotting. For quantification of conjugated and unconjugated UFC1 coloc2 software implemented in ImageJ was used. (**E**) Western blot of the peaks of the FL$^{1-404}$ C250K~UFM1 or AD$^{1-330}$ C250K~UFM1 gel filtration (α-UFM1). The corresponding protein bands are marked.

Table S1. NMR and refinement statistics for the UFC1:UBA5 R3$^{381–404W}$ complex.

**NOE assignment**	
Total NOE	11131
Assigned NOE	9850
% assigned	88.5
**NMR distance and dihedral constraints**	
Distance constraints	
Total NOE	4465
Intra-residue ($i = j$)	994
Sequential ($\lvert i - j \rvert = 1$)	969
Medium-range ($1 < \lvert i - j \rvert < 5$)	1094
Long-range ($\lvert i - j \rvert \geq 5$)	1408
Intermolecular	344
Hydrogen bonds	0
Total dihedral angle restraints	
ϕ	179
ψ	189
**Ramachandran plot**	
Residues in most favored regions	82.6%
Residues in additionally allowed regions	17.2%
Residues in generously allowed regions	0.1%
Residues in disallowed regions	0%
**Structure statistics**	
Violations (mean and s.d.)	
Distance constraints (Å)	0.0100 ± 0.003
Dihedral angle constraints (°)	0.37 ± 0.03
Max. dihedral angle violation (°)	3.83
Max. distance constraint violation (Å)	0.12
Deviations from idealized geometry	
Bond lengths (Å)	0.011 ± 0.002
Bond angles (°)	2.1 ± 0.08
Average r.m.s. deviation to mean (20 structures, Å)	
Heavy atoms of residues 3–162, 382–404	0.75 ± 0.06
Backbone atoms of residues 3–162, 382–404	0.37 ± 0.04

Supplementary references

1. Mizushima, T.; Tatsumi, K.; Ozaki, Y.; Kawakami, T.; Suzuki, A.; Ogasahara, K.; Komatsu, M.; Kominami, E.; Tanaka, K.; Yamane, T. Crystal structure of Ufc1, the Ufm1-conjugating enzyme. *Biochem. Biophys. Res. Commun.* **2007**, *362*, 1079-1084, doi:https://doi.org/10.1016/j.bbrc.2007.08.129.
2. Liu, G.; Forouhar, F.; Eletsky, A.; Atreya, H.S.; Aramini, J.M.; Xiao, R.; Huang, Y.J.; Abashidze, M.; Seetharaman, J.; Liu, J. NMR and X-RAY structures of human E2-like ubiquitin-fold modifier conjugating enzyme 1 (UFC1) reveal structural and functional conservation in the metazoan UFM1-UBA5-UFC1 ubiquination pathway. *J. Struct. Funct. Genomics* **2009**, *10*, 127-136, doi:https://doi.org/10.1007/s10969-008-9054-7.
